# Evolutions of Cube ({001}<100>) and {115}<161> Orientations in Cold-Rolled Ultra-Thin Non-Oriented Silicon Steel

**DOI:** 10.3390/ma16206735

**Published:** 2023-10-17

**Authors:** Yang Tu, Li Meng, Ning Zhang, Jiangjie Xu

**Affiliations:** Metallurgical Technology Institute, Central Iron and Steel Research Institute Co., Ltd., Beijing 100081, China; ty0171123688@163.com (Y.T.); zhangning@cisri.com.cn (N.Z.); jiejie051618@163.com (J.X.)

**Keywords:** ultra-thin non-oriented silicon steel, crystal plasticity finite element method (CPFEM), cold rolling, cube orientations ({001}<100>), {115}<161> orientations

## Abstract

In this study, the evolutions of Cube and {115}<161> orientations of a cold-rolled ultra-thin non-oriented silicon steel were investigated using a combination of experimental investigation and the crystal plasticity finite element method (CPFEM). The results show that Cube orientations remain relatively stable when their initial deviation angles from the ideal Cube orientation are less than 12°, even after a 60% cold rolling reduction. However, larger deviations occur due to higher strain near grain boundaries. Furthermore, the {115}<161> orientations, with an initial deviation of ~18° from the ideal Cube orientation, become separated into different orientation regions during cold rolling. Some regions gradually approach the ideal Cube orientation as cold rolling progresses and reach ~12.5° deviation from the ideal Cube at a 40% reduction. This study demonstrates good agreement between simulation and experimental results, highlights the micro-deformation mechanisms during rolling, and offers insights for optimizing the ultra-thin strip rolling process.

## 1. Introduction

As an excellent soft magnetic metal material, non-oriented silicon steel is widely used as the core material of motors and generators. With the rapid development of high-speed, miniaturization, and low-loss motors, non-oriented silicon steel requires higher magnetic induction intensity to enhance motor torque and miniaturize motor size, as well as lower core losses to improve motor efficiency [1,2,3,4,5,6]. The classical eddy current loss is proportional to the square of both the operating frequency and the sheet thickness, which dominates the overall core loss at high frequencies. Reducing the thickness of silicon steel is the most effective way to reduce iron loss and volume [7]. However, with the increase in cold rolling reduction, the γ-fiber texture (<111>//ND or {111}⊥ND) components gradually increase, and it is difficult to take into account both high magnetic induction and low iron loss. The magnetic properties of silicon steel are strongly dependent on texture and microstructure. Therefore, the optimization of the recrystallization texture of ultra-thin non-oriented silicon steel is an effective means to solve the problem of high magnetic induction and low iron loss, that is, to strengthen the λ-fiber texture (<001>//ND or {001}⊥ND), which is beneficial to the magnetic properties of non-oriented silicon steel, and to suppress the γ-fiber texture (<111>//ND or {111}⊥ND), which is not conducive to the magnetic properties.

A great deal of work has been carried out by scholars on how to enhance the λ-fiber texture and suppress the γ-fiber texture. Zhang et al. [8,9,10,11] observed the heritability of λ-fiber texture during cold rolling and annealing of the initial microstructure of columnar crystals. Li et al. [12] reported that thin strips with a higher content of λ-fiber texture by twin-roll continuous casting can obtain strong {001} recrystallization texture after appropriate rolling and heat treatment, with γ-fiber recrystallization texture almost disappearing, with higher magnetic induction and lower iron loss, reflecting the genetic law of λ-fiber texture. Qin et al. [13] indicated that the microstructure with strong {100}<012>-{411}<148> recrystallization texture can obtain strong {001} recrystallization texture and weak γ-fiber recrystallization texture after medium cold rolling reduction and annealing. An et al. [14] identified that increasing the grain size of non-oriented silicon steel before finally cold rolling can promote the formation of shear bands during cold rolling, resulting in the enhancement of λ-fiber texture and the weakening of γ-fiber texture after annealing. Lan et al. [15] proposed that thin strips made by twin-roll continuous casting through an annealing-cold rolling-annealing process have a stronger λ-fiber texture and a lower γ-fiber texture than those fabricated by a direct cold rolling-annealing process. Zhang et al. [16] and Jiao et al. [17] prepared non-oriented silicon steel with the two-stage cold rolling process. Compared with the one-stage cold rolling process, the two-stage cold rolling process weakens the unfavorable γ-fiber texture, enhances the λ-fiber texture of the final sheet, and improves the magnetic properties of the product. The recrystallization texture of non-oriented silicon steel is closely related to the initial texture and cold rolling texture.

With the rapid development of computer technology and numerical methods, it is more and more common to study the evolution of the microstructure of metal materials during deformation by using the crystal plasticity finite element method. Chu et al. [18] reported the orientation change and the accumulation of deformation energy storage of different initial texture components during cold rolling of non-oriented silicon steel by combining crystal plasticity finite element simulation and experiments. They pointed out that the accumulation rates of energy storage during cold rolling deformation of initial λ orientations were significantly lower than those of γ and α orientations, and even if different initial orientations were transferred to the same deformation orientations, the accumulation of strain energy storage would be different. Klusemann et al. [19] studied the deformation behavior of Fe-3% Si under experimental tension by using the crystal plasticity finite element method, and the results were in good agreement with the experimental results.

Ultra-thin non-oriented silicon steels with a thickness <0.10 mm generally choose the commercially thick specification of non-oriented silicon steel as the initial material (base material); they can be used as core materials to meet medium- and high-frequency operating conditions after precision cold rolling and annealing treatment. Improving magnetic induction and reducing medium- and high-frequency losses are important goals for ultra-thin, non-oriented silicon steel. However, there have been few reports on the microstructure and textures of the initial material and the cold rolling behavior for the preparation of ultra-thin, non-oriented silicon steel with a thickness of <0.10 mm. In this paper, commercial high-performance non-oriented silicon steels with Cube texture, near-cube texture, and Goss texture as the main texture characteristics are used as raw materials to study the evolution of textures during cold rolling. The Cube texture has attracted special attention because it is beneficial to the rolling direction and transverse magnetic induction of silicon steel rolling sheets. In the conventional silicon steel system, the Cube orientations generally rotate toward the α-fiber along the path. Cube-{001}<011> during rolling deformation [20]; Jiao et al. [21] indicated that the Cube orientations rotate frequently toward Cube-{001}<130>-{001}<120> and occasionally along the paths. Cube-{013}<031>-{012}<021>-{110}<110> and Cube-{001}<130>-{114}<481>-{112}<241> et al. in Fe-1.3 wt% Si under large cold rolling reductions. Zhang et al. [22] proposed that there is a Cube-{001}<130>-{113}<251> transition path during the cold rolling deformation of columnar grains. 

So far, in the ultra-thin, non-oriented silicon steel system, there have been few reports on the cold-rolled evolution law of the initial Cube and near-cube textures. Therefore, this paper adopts a combination of CPFEM simulation and experiments to investigate the evolution of the microstructure and textures of Cube and near-cube-oriented grains in the base metal of initial non-oriented silicon steel after cold rolling, which is expected to provide a basis for the control of cube texture. In addition to grain orientations, microstructure parameters such as initial grain size and grain boundaries were considered in the simulation. The cumulative slip distributions, slip distributions of different slip systems, and the transition laws of grain orientations were analyzed. Compared with other studies, by clarifying the rotation routes of Cube and near cube orientations, this work focuses on the cold rolling evolutions of Cube and {115}<161> (near Cube) orientations, and detailed information about the critical deviation degree of cube orientation from ideal Cube to remain stable, as well as the behavior of {115}<161> orientation transiting towards cube orientation, was suggested, while the microstructure change inside the grain was discussed. This work could also provide theoretical guidance for optimizing the rolling process of ultra-thin, non-oriented silicon steel strips and exploring the deformation mechanism during rolling deformation. 

## 2. Materials and Methods

### 2.1. Experimental Materials and Characterization Methods

**Figure 1:**
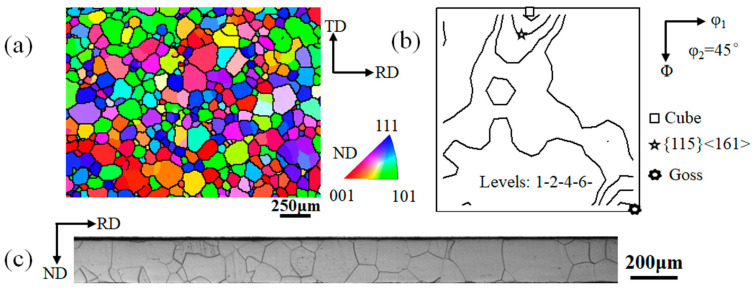
The initial sample was commercial 0.20 mm-thick non-oriented silicon steel. (**a**) EBSD mapping of the inverse pole figure (IPF); (**b**) φ_2_ = 45° ODF section; (**c**) Microstructure.

The commercial high-performance non-oriented silicon steel with 0.2 mm was used as the base material, as shown in Figure 1. There are relatively sharp Cube textures, {115}<161> texture, and Goss texture in Figure 1b, in which the {115}<161> component deviates from the ideal Cube by 18°, and the average grain size is about 100 μm in Figure 1a. The initial sample was subjected to multi-pass cold rolling to 0.08 mm (cumulative reductions of ~60%). The samples with a size of 10 mm (RD) × 5 mm (TD) × 0.08 mm (ND) were intercepted from the cold-rolled samples. The HKL Nordlys Max3 EBSD (UK High Wycombe Oxford Instrument Technology Co., Ltd., Oxford, UK) detector equipped with the GeminiSEM 500 (Carl Zeiss Management Co., Ltd., Oberkochen, Germany) field emission scanning electron microscope was used to collect the morphology and orientation data with different cold rolling reductions. The HKL Channel 5 software (version 5.11.20405.0) was used to obtain the orientation imaging and orientation distribution data. Due to the large initial grain size, to express the statistical significance of the experimental results, multiple non-adjacent regions were selected for EBSD characterization and comprehensive analysis of all samples. In this paper, only representative data were listed. The maximum deviation angle of Cube orientations was set to 10°, the {001}<130> orientations were set to 9°, and the {115}<161> orientations were set to 8°. The CPFEM was carried out by ABAQUS software (version 2016), the polar diagrams were drawn, and the misorientations were calculated by plug-in MTEX in the software MATLAB (version 2021a). 

### 2.2. CPFEM and Model Establishment

The CPFEM on phenomenology is used to simulate the deformation behavior under different cold rolling reductions. Phenomenology includes the deformation gradient decomposition theory and the crystal plastic constitutive model [23]. The total deformation gradient F can be decomposed as

F = F* · F^P^, (1)

where F* is the elastic deformation gradient tensor and F^P^ is the plastic deformation gradient tensor. The crystal plasticity constitutive model adopted the hardening model proposed by Asaro et al. [24,25,26]. Based on the Schmid law, the shear strain rate ${\dot{\gamma }}^{\left(\alpha \right)}$ of the αth slip system is determined by the corresponding resolved shear stress $\tau^{\left(\alpha \right)}$**,** for any slip system:

(2) $${\dot{\gamma }}^{\left(\alpha \right)}={{\dot{\;\gamma }}_{0}}^{\left(\alpha \right)}\mathrm{sgn}\left(\tau^{\left(\alpha \right)}\right){\left|\frac{\tau^{\left(\alpha \right)}}{g^{\left(\alpha \right)}}\right|}^{n},$$

(3) $${\dot{g}}^{\left(\alpha \right)}=\sum_{\beta }h_{\alpha \beta }\;{\dot{\gamma }}^{\left(\beta \right)},$$

(4) $$h_{\alpha \beta \;}=\;qh_{\alpha \alpha },\;\;\;\alpha \;\neq \;\beta ,$$

(5) $$h_{\alpha \alpha }=h_{0}{\sec h}^{2}\left(\frac{h_{0}\gamma }{\tau_{s}\;-\;\tau_{0}}\right),$$

(6) $$\gamma =\sum_{\alpha }^{N}\int_{0}^{t}\left|{\dot{\gamma }}^{\left(\alpha \right)}\right|\mathrm{dt},$$

where the ${{\dot{\gamma }}_{0}}^{\left(\alpha \right)}$ is the reference strain rate on slip system α, $g^{\left(\alpha \right)}$ is a variable that describes the reference strength of that system, $\mathrm{sgn}\left(\tau^{\left(\alpha \right)}\right)$ is a general non-dimensional function that describes the dependence of strain rate on the stress, n is the rate sensitivity exponent, and in the limit as n→∞ this power law approaches that of a rate-independent material, ${\dot{g}}^{\left(\alpha \right)}$ is the evolution of the strengths $g^{\left(\alpha \right)}$, $\;{\dot{\gamma }}^{\left(\beta \right)}$ is the slipping rate of the βth slip system, $h_{\alpha \beta }$ is the latent hardening modulus, $h_{\alpha \alpha }$ is the self-hardening modulus, $h_{0}$ is the initial hardening modulus, q is the ratio of latent hardening modulus to self-hardening modulus, $\tau_{s}$ is the stage I stress, $\tau_{0}$ is the yield stress, γ is the Taylor cumulative shear strain on all slip systems. The crystal plasticity constitutive model was written into the user subroutine UMAT of ABAQUS (version 2016) for the subsequent simulation process. The non-oriented silicon steel used in this experiment is a polycrystalline ribbon with a single BCC crystal structure, with 24 slip systems mainly {110}<111> and {112}<111>, and with slip surfaces and slip directions as shown in Table 1. The elastic constants are C_11_ = 226 GPa, C_12_ = 140 GPa, and C_44_ = 116 GPa [27,28], and the remaining hardening parameters are obtained by fitting the stress-strain curve obtained from the tensile test of the base metal, as shown in Table 2.

**Figure 2:**
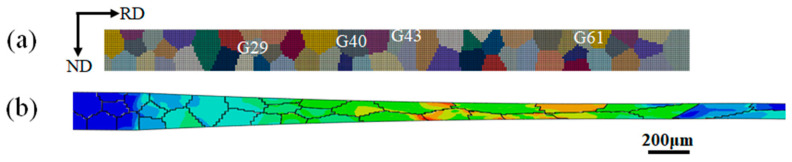
Finite element model verification demonstration. (**a**) Reconstructed initial microstructure-different colors represent different orientations; (**b**) Microstructure after rolling-different colors represent different shape variables.

The grains in the polycrystalline body traverse the gap between the upper and lower rolls and undergo plastic deformation during the cold rolling deformation. After cold rolling deformation, the rolled material is elongated along the rolling direction, thinned along the normal direction, and basically unchanged along the transverse direction [7]. Therefore, the rolling process of non-oriented silicon steel strips is simplified to plane strain compression. The more representative-oriented grains were randomly selected in the grain structure obtained by the EBSD technique. Based on the Voronoi method, the polycrystalline model was reconstructed, and the measured grain orientations were given. In the finite element model of ABAQUS, the calculation model size was 3.5 mm (RD) × 0.2 mm (ND), and the CPE4R unit was used to discretize the model, which was 7000 units. The verification demonstration of the discrete finite element model is shown in Figure 2. Different colors correspond to different grain orientations, and the unit aggregate with the same orientations is a grain, denoted as Gn.

## 3. Results and Discussion

### 3.1. Microstructure Evolutions of Different Cold Rolling Reductions

**Figure 3:**
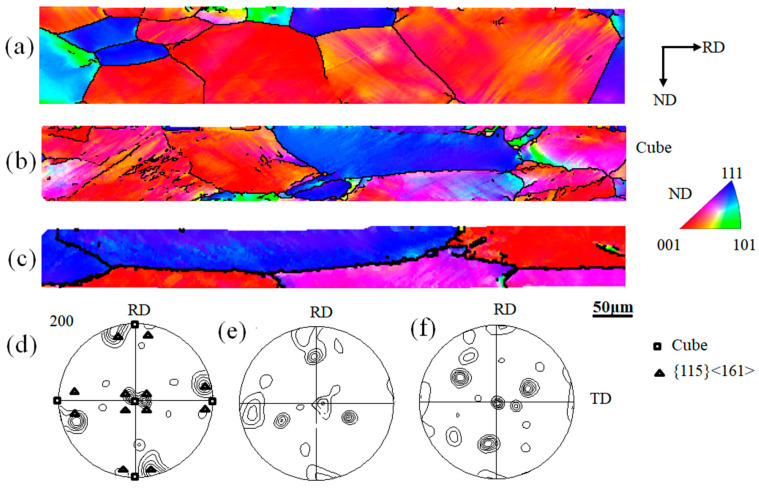
EBSD mapping of inverse pole figure (IPF) and {200} pole figures of 0.2 mm-thick non-oriented silicon steel with cold rolling reductions of (**a**,**d**) 20%, (**b**,**e**) 40%, and (**c**,**f**) 60%.

Figure 3 shows the IPF map and {200} pole figures of non-oriented silicon steel at different cold rolling reductions. With the increase in cold rolling reductions, the microstructure is gradually elongating. The specimen with a 20% reduction in Figure 3a,d mainly displays a near Cube texture, and a Cube texture can also be observed. When the rolling reduction is 40%, the γ-fiber texture appears in Figure 3b,e; however, the existence of near Cube texture and Cube texture can still be found. As the reduction increases to 60%, Figure 3c,f shows that the texture is mainly composed of γ-fiber texture and Cube texture. Therefore, for this system: (1) at 40% cold rolling reduction, the Cube texture and the near Cube texture appear simultaneously, and the near Cube {115}<161> orientations can be observed near the Cube orientations; (2) The Cube texture can still be partially retained even after 60% moderate reduction; (3) when the cold rolling reduction is greater than 40%, the appearance of γ-fiber texture can be observed. 

### 3.2. Evolutions of Cube and {115}<161> Orientations during Cold Rolling

**Figure 4:**
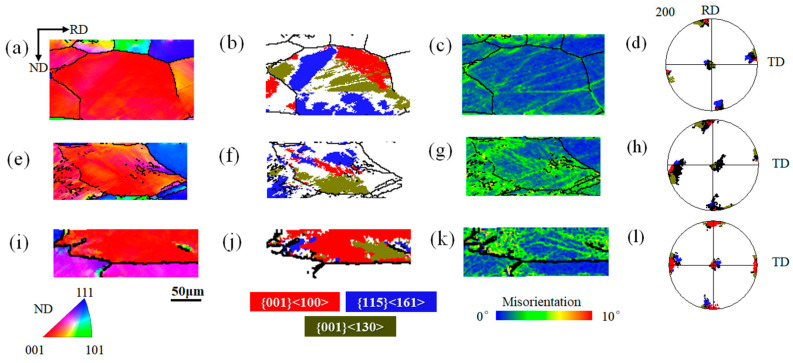
Microstructure and orientation distributions of Cube-oriented and near-cube-oriented grains at different cold rolling reductions. Grain 1 with 20% Cold rolling reduction: (**a**) EBSD mapping of inverse pole figure (IPF), (**b**) orientation image map (OIM), (**c**) local misorientation (LM) map, (**d**) {200} pole figures. Grain 2 with 40% Cold rolling reduction: (**e**) IPF map, (**f**) OIM map, (**g**) LM map, (**h**) {200} pole figures. Grain 3 with 60% Cold rolling reduction: (**i**) IPF map; (**j**) OIM; (**k**) LM map; (**l**) {200} pole figures.

Cube and near-cube-oriented grains with different cold rolling reductions in Figure 3 are selected, and their microstructure and orientation distributions are shown in Figure 4. When the cold rolling reduction is 20%, Figure 4a,d shows that grain 1 is near Cube orientation, and there are orientation gradient distributions inside; however, Figure 4c shows the low local misorientations among Cube, {001}<130>, and {115}<161> orientations. The Cube, {001}<130>, and {115}<161> orientation regions are distributed inside the grain in Figure 4b, where there is a diffuse cross-distribution between the Cube and {001}<130> orientation regions. Combined with the rotation law of grain orientations during cold rolling, it can be seen that the {001}<130> orientations can come from the rotation of the Cube orientations around the <001>//ND axis during cold rolling, and the rotation trend is usually considered irreversible. Since the deformation of the grain is relatively slight at 20% deformation, most of the initial characteristics of the grain deviate less. It can be seen from Figure 4d that there is a rotational relationship between the Cube orientations and the {115}<161> orientations. When the cold rolling reduction is 40%, the {001}<130> orientations in grain 2 come from the rotation of the Cube orientations during cold rolling. It can be seen from Figure 4f that the Cube orientations are distributed between the {115}<161> orientations and the {001}<130> orientations. Figure 4g shows that there is an orientation gradient between the three regions. According to the {200} pole figure of Figure 4h, combined grain 1, it is guessed that there is a {115}<161>-Cube-{001}<161> rotation path inside grain 2. When the cold rolling reduction reaches 60%, Figure 4j shows that the internal orientations of grain 3 are mainly concentrated in the Cube orientations, and the {001}<130> orientation regions are distributed in blocks, which is speculated to be derived from the rotation of the initial Cube orientations during the cold rolling deformation process, and the {115}<161> orientation regions are dispersed. Figure 4k shows that the local misorientations in the {001}<130> regions are smaller, while the Cube and {115}<161> regions are larger. Combined with Figure 4l, it is conjectured that the initial orientations of grain 3 may be {115}<161> orientations, and a large number of Cube orientations are formed during cold rolling deformation. The existence of a large number of Cube orientation regions shows that the Cube orientations can still maintain certain stability at 60% deformation. 

In summary, both Cube and {115}<161> orientations are simultaneously observed at different cold rolling deformations and inside different grains. However, due to the limitations of experimental conditions, the rotation relationship between Cube orientations and {115}<161> orientations cannot be completely determined. Moreover, it is found that Cube orientations still exist inside some grains at a moderate deformation of 60%. However, the source and stable existence conditions of Cube orientations are still unclear. Therefore, the evolutions of Cube orientations from the ideal Cube for different deviation angles and {115}<161> orientations of different cold rolling reductions are simulated by the CPFEM. 

### 3.3. Simulation of Cold Rolling Behaviors of Cube and {115}<161> Orientations

#### 3.3.1. Cumulative Slip Distributions, Orientation Evolutions, and the Distributions of Different Slip System Activation of Cube Orientations with Different Cold Rolling Reductions

**Figure 5:**
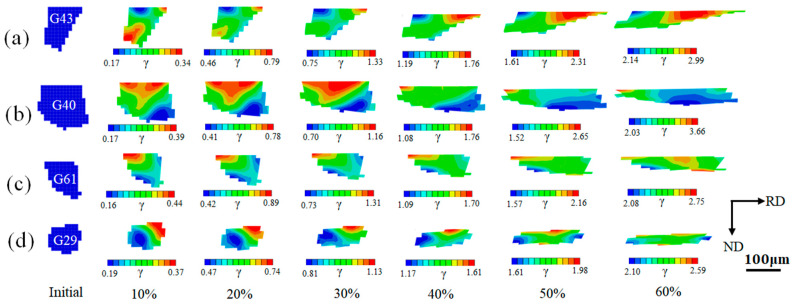
Cumulative plastic slip distributions of grains of Cube orientations with different deviation angles from the ideal Cube orientation at different cold rolling reductions. (**a**) G43: deviation angle is 2.6°; (**b**) G40: deviation angle is 5.8°; (**c**) G61: deviation angle is 8.9°; (**d**) G29: deviation angle is 12.2°.

The initial morphology of the grain was reconstructed through the Voronoi method, and the grain size was expressed by the number of grid cells contained in the grain. Here, the grains G43, G40, G61, and G29, which deviate from the ideal cube orientation at different angles, are selected as the research objects. The grain G43 contains 90 cells, G40 contains 174 cells, G61 contains 68 cells, and G29 contains 70 cells. The grain orientations coming from the EBSD measured were randomly assigned to different grain sizes in the simulation. The cumulative plastic slips reflect the sum of the slips of the activated slip system of the 24 slip systems in the rolling deformation process [29], and their distribution state along the thickness direction and the rolling direction of the thin strip reflects the non-uniformity of the micro-deformation of the ultra-thin strip rolling. It can be seen that the high-strain slip zones and the low-strain slip zones inside the grain are basically distributed at the grain boundaries in Figure 5. Due to the coordination of adjacent grains and the coordinated deformation inside the grain, the grain boundaries are prone to slip and hinder the trend, which is obviously different from the micro-deformation inside the grain. With the increase in rolling reductions, the degree of slip inside the grain gradually increases. The evolution of the cumulative slip in the deformation is non-uniform, and the phenomenon of non-uniform localization gradually increases. There are many non-uniform deformation zones in the grain. This phenomenon of deformation inhomogeneity is particularly obvious at higher cold rolling reductions with larger grain sizes. The difference between the high and low strain zones of G40 grain at 50% and 60% cold rolling deformation is significantly larger than that of other grains, and the local inhomogeneity inside the grain increases. It can be seen that the smaller grain size can weaken the localization trend of slip [30]. The larger the grain size is, the higher the cumulative slip degrees are inside the grain, and the easier it is to form shear bands during cold rolling deformation. Figure 5b shows that when the deformation of the G40 grain is greater than 50%, multiple slip bands are formed inside the grain. 

**Figure 6:**
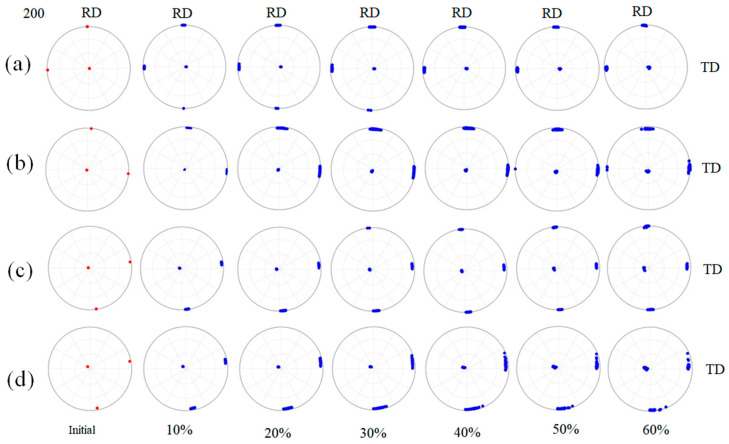
Orientation evolutions of Cube orientations with different deviation angles from the ideal Cube orientation at different cold rolling reductions—red represents the initial orientation, and blue represents the orientation after cold rolling. (**a**) G43: deviation angle is 2.6°; (**b**) G40: deviation angle is 5.8°; (**c**) G61: deviation angle is 8.9°; (**d**) G29: deviation angle is 12.2°.

**Figure 7:**
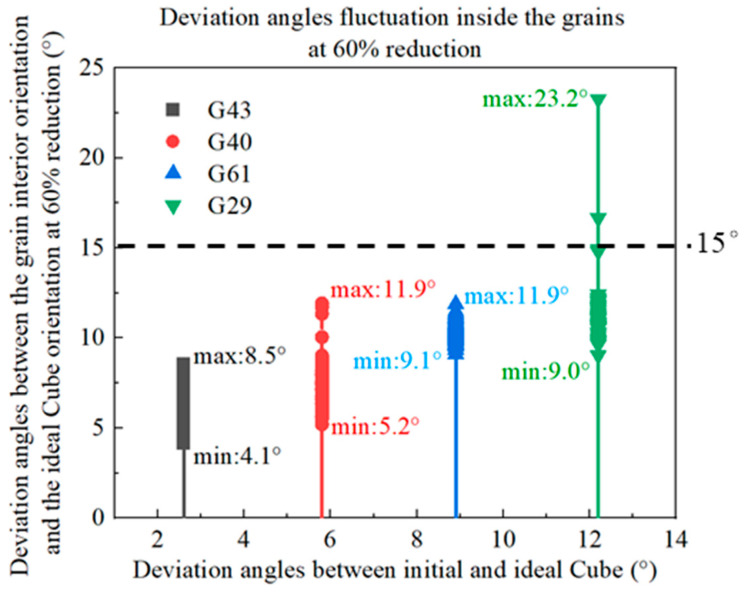
Distributions of the deviation angles between the internal orientations of different grains and the ideal Cube orientation at 60% cold rolling reduction.

Figure 6 shows the orientation evolutions of Cube orientations with different deviation angles from the ideal Cube orientation during cold rolling deformation. In the figure, red represents the initial orientation, and blue represents the orientation after cold rolling. It can be seen that with the increase in cold rolling reductions, the grain orientations rotate and present a discrete trend in the grain in Figure 6, which corresponds to the inhomogeneity of the cumulative slip inside the grain in Figure 5. During the cold rolling process, parts of the cube rotate around the ND//<001> axis. With the increase in cold rolling reductions, the rotation angles of grain orientations gradually increase; however, the overall rotation amplitude is still small. Figure 7 displays the fluctuation distributions of the deviation angles between the internal rotation orientations and the ideal Cube orientation of four kinds of Cube-oriented grains with different degrees of deviation at 60% reduction. When the deviation angles between the initial grain orientations and the ideal Cube orientation are less than 10°, at a deformation of 60%, the grain orientations rotate but the rotation amplitudes are smaller. Combining Figure 5 and Figure 7, it can be found that the dispersion of deviation angles and the deformation inhomogeneity inside G43 and G61 grains are smaller than the G40 grain, which is related to the initial grain size. The above three orientations still do not deviate from the ideal Cube orientation by more than 15° at 60% cold rolling reduction. When the initial grain orientations deviate from the ideal Cube orientation by an angle of 12.2°, a few of the region orientations within the G29 grain show a large deviation of the orientations (away from the ideal Cube orientation), with the maximum deviation angle reaching ~23°. Therefore, we speculate that when the deviation angles between the initial orientations and the ideal Cube orientation are less than 12°, the Cube orientations can maintain certain stability at a moderate reduction. This stability is related to the grain size. The smaller the grain size is, the better the stability is, as shown in Figure 5. In addition to this, this may be due to the accumulated slip caused by different activated slip systems. For the cube orientation with low deviation, it is suggested that the lower number of activated slip systems and the lower degrees of slip of each activated slip system lead to fair stability, and this will be discussed in more detail later.

**Figure 8:**
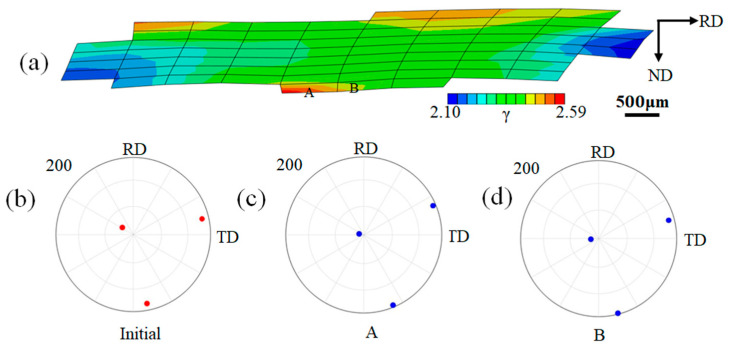
Microstructure of G29 grain and {200} pole figures of the local area with 60% reduction—red represents the initial orientation, and blue represents the orientation after cold rolling. (**a**) Cumulative plastic slip distributions; (**b**) {200} pole figure of the initial state; (**c**) {200} pole figure of A; (**d**) {200} pole figure of B.

For the G29 grain that appears to have a few orientations with a large orientation deviation from the ideal Cube orientation, Figure 8 shows the simulated cumulative slips of each region and calculates the {200} pole figures of the local regions at 60% reduction. It can be seen from Figure 8 that the maximum slip degree of the grain is at A, and the slip degree at B is also large. The strain is higher near the grain boundaries due to the generation of statistically stored dislocations. Figure 8c shows the orientation after deformation at A, with a deviation angle of ~23° from the ideal Cube orientation; Figure 8d shows the orientation of B after deformation, and the deviation angle from the ideal Cube orientation is ~16°. 

**Figure 9:**
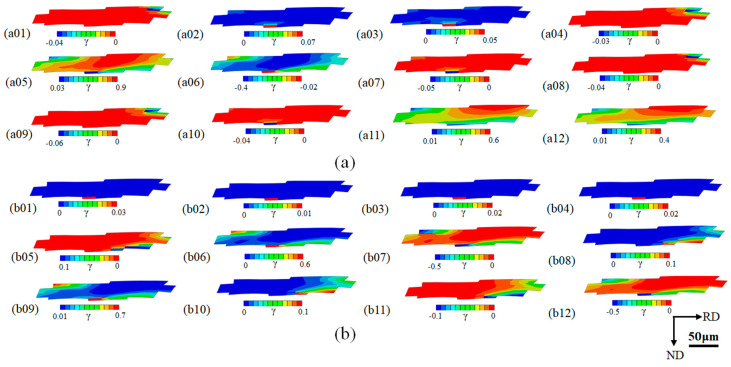
The slip distributions of 24 slip systems of G29 grain were reduced by 60%. (**a**) a01–a012, (**b**) b01–b012.

Figure 9 shows the slip distributions of 24 slip systems of G29 grain with a 60% reduction, with a negative value indicating that the slip direction is opposite to the direction of the prescribed slip systems (Table 1). It can be seen that during the cold rolling deformation process, the number of activated slip systems and the degrees of slip of each activated slip system at different locations within the grain are different. The maximum number of activated slip systems and the maximum slip degree of activated slip systems are basically distributed at the grain boundaries, which is related to the coordinated deformation of the surrounding grains and inside the grain. During the process of cold rolling deformation, 16 slip systems are activated at A, among which b06, b09, and b12 slip systems have the greatest degree of slip at A, while a02, a03, a07, a10, b01, b02, b03, and b04 slip systems, although with a lower degree of slip, can be seen from Figure 9 that these slip systems are basically activated only at A during the cold rolling deformation, and other regions inside the G29 grain are not activated. Therefore, the cumulative slips of most activated slip systems result in the largest slip degree and the largest orientation rotation angle at A. In addition to A, the number of activated slip systems at other grain boundaries (such as B in Figure 8a) of G4 grain is also large, as is the degree of slip, and the orientation also produces a large deviation angle during rotation, attributing to the generation of statistically stored dislocations and resulting in large strains. Since the rotation speed used in the simulation is consistent with the actual roll speed, the deformation of the grains is almost instantaneous. During the deformation process of the grains, the slip system is a unidirectional slip from start to stop. 

#### 3.3.2. Cumulative Slip Distributions and Evolutions of {115}<161> Orientations of Different Cold Rolling Reductions

**Figure 10:**
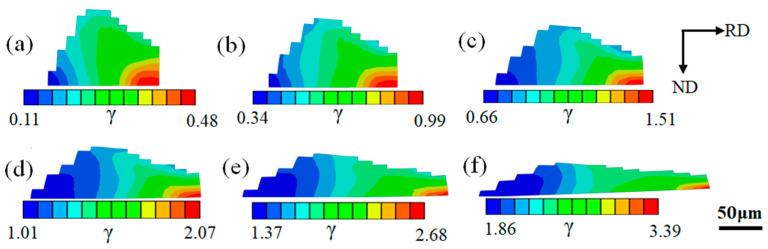
Cumulative plastic slip distributions of {115}<161> oriented grain with cold rolling reductions of (**a**) 10%, (**b**) 20%, (**c**) 30%, (**d**) 40%, (**e**) 50%, and (**f**) 60%.

Based on the experimental results, we simulated the evolutions of the {115}<161> orientations of different cold rolling reductions, which are adjacent to the cube orientations many times and contained in the initial texture type. Figure 10 shows the cumulative plastic slip distributions of {115}<161> oriented grain with different cold rolling reductions. It can be seen that with the increase in cold rolling reductions, the degrees of slip and the non-uniform localization phenomenon inside the grain gradually increase, and there are obvious multiple slip bands throughout the grain thickness. Compared with Figure 5, the local slip inhomogeneity inside the {115}<161> oriented grain is stronger when the grain size is the same.

**Figure 11:**
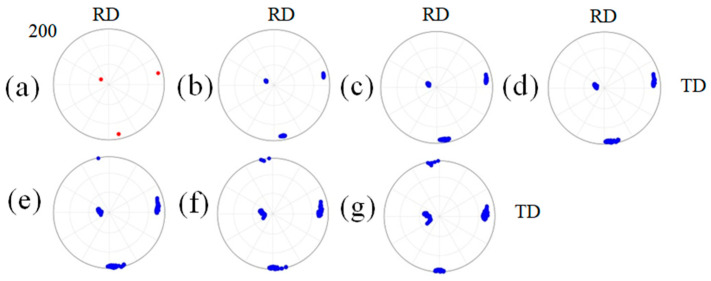
Orientation evolutions of {115}<161> orientations with cold rolling reductions of (**a**) initial, (**b**) 10%, (**c**) 20%, (**d**) 30%, (**e**) 40%, (**f**) 50%, and (**g**) 60%. Red represents the initial orientation, and blue represents the orientation after cold rolling.

**Figure 12:**
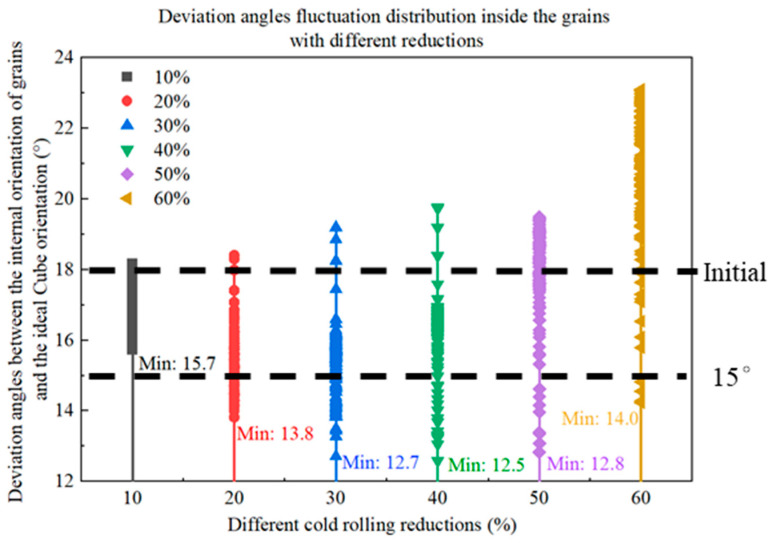
Distributions of the deviation angles between the internal orientations of {115}<161> oriented grain and the ideal Cube orientation at different cold rolling reductions.

Figure 11 shows the orientation evolutions of {115}<161> orientations during cold rolling deformation. It can be seen that with the increase in cold rolling reductions, the orientation and rotation angles inside the grain gradually increase. The internal orientations of the grain will “separate” from several different orientation regions during the cold rolling process. Combined with Figure 10, it can be seen that the difference in the slip degrees of slip systems in each region inside the grain leads to the grain "“fragmentation”. The regions of “fragmentation” are separated by transition bands, that is, multiple slip bands, as shown in Figure 10. Each region separated is called a cell block, and the large deviation angles between the cell blocks are caused by the different slip degrees of the slip systems. The formation of multiple slip bands indicates that the fluctuation distributions of the deviation angles within the grain are relatively large, as shown in Figure 12. When the cold rolling reduction is greater than 40%, the difference in orientation dispersion inside the grain is more obvious (Figure 11). When the cold rolling reduction is less than 40%, the internal orientations of the grain mainly rotate around the ND axis. Combined with Figure 12, it can be seen that most of the orientations inside the grain gradually move from the initial {115}<161> orientations (~18° from the ideal Cube orientation) to the Cube orientations. The orientations of some regions are shifted to ~12.5° from the ideal Cube orientation at 40% cold rolling reduction; however, they are still separated from the orientation micro-regions whose orientations deviate more than 12.5° with the increase in cold rolling reductions. Despite all this, there are still some orientation regions with deviation angles of <15° from the ideal Cube orientation inside the grain at 60% reduction. Compared with the experimental results, it can be inferred that there may be a rotation path near Cube {115}<161> toward Cube during the rolling process; that is, some of the Cube orientations in the previous experimental diagram may come from the rotation of {115}<161> orientations and maintain a certain stability in the subsequent.

## 4. Conclusions

The evolutions of the cold-rolled Cube and {115}<161> orientations with different cold-rolling reductions were investigated by combining experiments with the CPFEM. The cumulative plastic slips and grain orientation rotations during the deformation process were analyzed, which provides certain theoretical guidance for optimizing the rolling process of ultra-thin strips and exploring the deformation mechanism during rolling deformation.

(1) The CPFEM is used to simulate the evolutions of Cube and {115}<161> orientations under different cold rolling reductions. The cumulative slip distributions, slip distributions of different slip systems, rotations of grain orientations, and grain deviation angles within the grains of different orientations on mesoscopic scales were calculated. Through the CPFEM, the mechanism of the influence of the cold rolling process on the micro-deformation of the grains in the ultra-thin non-oriented silicon steel was obtained, which provides some theoretical guidance for the optimization of the ultra-thin strip rolling process and the exploration of the deformation mechanism in the deformation process of the rolling.
(2) Cube orientations in the initial material with less than 10° deviation from the ideal Cube orientation are still less than 15° deviation from the ideal Cube orientation even after 60% cold rolling deformation. The smaller the grain size is, the more uniform the cumulative slip distributions are within the grain, and the smaller the degrees of orientation dispersion inside the grain are. When the initial orientations do not deviate from the ideal Cube orientation by more than 12°, and the cold rolling reduction reaches 60%, the angles of deviation from the ideal Cube orientation in the grain center region are still less than 15°, indicating that the Cube orientations have a certain degree of stability in cold rolling deformation, but near the grain boundaries, due to the large number of activated slip systems and the large degree of slip, statistically stored dislocations are generated, and the grain orientations are prone to deviate from the ideal Cube orientation by more than 15°.
(3) The grain with {115}<161> orientations deviating from the ideal Cube orientation by ~18° is “separated” into some different orientation regions during the cold rolling process, and some orientations of these regions are gradually oriented close to the ideal Cube orientation during the cold rolling process. When the cold rolling reduction reaches 40%, the orientations of some regions are shifted to ~12.5° from the ideal Cube orientation, which is still separated from the orientation micro-regions whose orientations deviate more than 12.5° with the increase in cold rolling reductions. There are still Cube orientation regions with an ideal Cube orientation of <15° inside the grain at 60% deformation.

## Figures and Tables

**Table 1:** Slip systems in BCC metals.

{110}<111>			{112}<111>		
No.	Slip Plane	Slip Direction	No.	Slip Plane	Slip Direction
a01	(011)	[$1\overset{-}{1}$1]	b01	(112)	[$11\overset{-}{1}$]
a02	(011)	[$11\overset{-}{1}$]	b02	($\overset{-}{1}$12)	[$1\overset{-}{1}$1]
a03	(101)	[$\overset{-}{1}$11]	b03	($1\overset{-}{1}$2)	[$\overset{-}{1}$11]
a04	(101)	[$11\overset{-}{1}$]	b04	($11\overset{-}{2}$)	[111]
a05	(110)	[$\overset{-}{1}$11]	b05	(121)	[$1\overset{-}{1}$1]
a06	(110)	[$1\overset{-}{1}$1]	b06	($\overset{-}{1}$21)	[$11\overset{-}{1}$]
a07	($0\overset{-}{1}$1)	[111]	b07	($1\overset{-}{2}$1)	[111]
a08	($0\overset{-}{1}$1)	[$\overset{-}{1}$11]	b08	($12\overset{-}{1}$)	[$\overset{-}{1}$11]
a09	($10\overset{-}{1}$)	[111]	b09	(211)	[$\overset{-}{1}$11]
a10	($10\overset{-}{1}$)	[$1\overset{-}{1}$1]	b10	($\overset{-}{2}$11)	[111]
a11	($\overset{-}{1}$10)	[111]	b11	($2\overset{-}{1}$1)	[$11\overset{-}{1}$]
a12	($\overset{-}{1}$10)	[$11\overset{-}{1}$]	b12	($21\overset{-}{1}$)	[$1\overset{-}{1}$1]

**Table 2:** Material parameters used in the simulation.

C_11_/GPa	C_12_/GPa	C_44_/GPa	n	$\dot{\gamma }$/s^−1^	$h_{0}$/MPa	$\tau_{s}$/MPa	$\tau_{0}$/MPa	q
226	140	116	20	0.001	240	1200	268	1 (1.4)

## Data Availability

Not applicable.
